# [(*Z*)-*N*-(4-Chloro­phen­yl)-*O*-methyl­thio­carbamato-κ*S*](triphenyl­phosphine-κ*P*)gold(I)

**DOI:** 10.1107/S1600536809050454

**Published:** 2009-11-28

**Authors:** Primjira P. Tadbuppa, Edward R. T. Tiekink

**Affiliations:** a Department of Chemistry, National University of Singapore, Singapore 117543; b Department of Chemistry, University of Malaya, 50603 Kuala Lumpur, Malaysia

## Abstract

The title compound, [Au(C_8_H_7_ClNOS)(C_18_H_15_P)], is characterized by a linear *S*,*P*-donor set with a small deviation from the ideal linearity [S—Au—P = 175.14 (5)°] due to the close approach of the O atom to the Au atom [Au⋯O = 2.882 (3) Å]. Loosely associated dimers are formed in the crystal structure through C—H⋯O inter­actions.

## Related literature

For structural systematics and luminescence properties of phosphinegold(I) carbonimidothio­ates, see: Ho *et al.* (2006 ▶); Ho & Tiekink (2007 ▶); Kuan *et al.* (2008 ▶). For the synthesis, see: Hall *et al.* (1993 ▶).
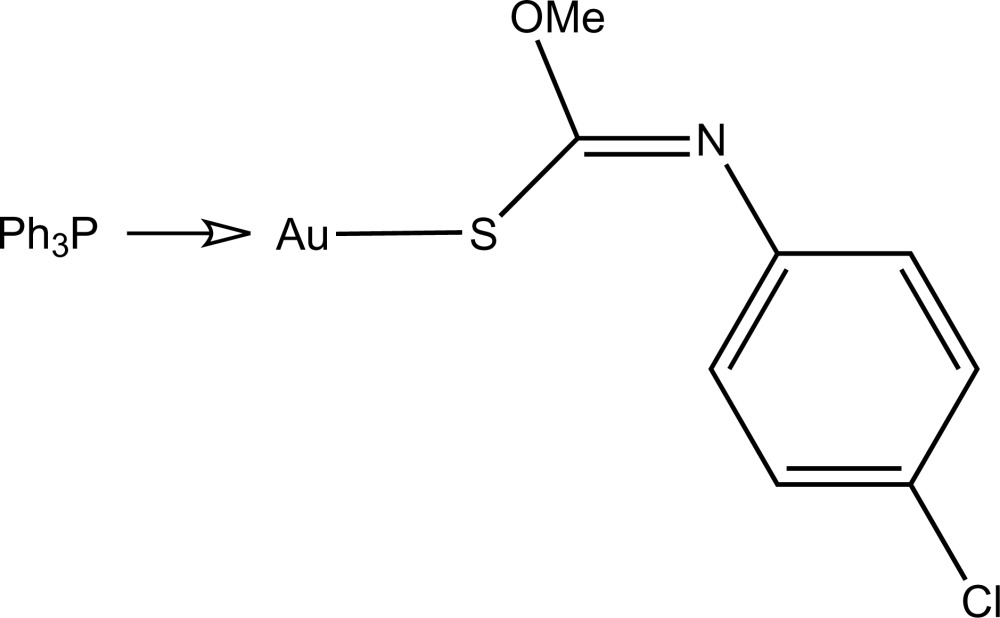


## Experimental

### 

#### Crystal data

[Au(C_8_H_7_ClNOS)(C_18_H_15_P)]
*M* *_r_* = 659.89
Monoclinic, 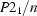

*a* = 9.1781 (6) Å
*b* = 17.5679 (12) Å
*c* = 15.5384 (11) Å
β = 104.156 (2)°
*V* = 2429.3 (3) Å^3^
*Z* = 4
Mo *K*α radiation
μ = 6.34 mm^−1^
*T* = 223 K
0.11 × 0.10 × 0.07 mm

#### Data collection

Bruker SMART APEX CCD diffractometer
Absorption correction: multi-scan (*SADABS*; Bruker, 2001 ▶) *T*_min_ = 0.363, *T*_max_ = 1.000
17091 measured reflections
5570 independent reflections
4526 reflections with *I* > 2σ(*I*)
*R*_int_ = 0.042

#### Refinement

*R*[*F* ^2^ > 2σ(*F* ^2^)] = 0.029
*wR*(*F* ^2^) = 0.090
*S* = 1.06
5570 reflections
290 parameters
H-atom parameters constrained
Δρ_max_ = 1.17 e Å^−3^
Δρ_min_ = −0.62 e Å^−3^

### 

Data collection: *SMART* (Bruker, 2007 ▶); cell refinement: *SAINT* (Bruker, 2007 ▶); data reduction: *SAINT*; program(s) used to solve structure: *PATTY* in *DIRDIF92* (Beurskens *et al.*, 1992 ▶); program(s) used to refine structure: *SHELXL97* (Sheldrick, 2008 ▶); molecular graphics: *DIAMOND* (Brandenburg, 1999 ▶); software used to prepare material for publication: *publCIF* (Westrip, 2009 ▶).

## Supplementary Material

Crystal structure: contains datablocks global, I. DOI: 10.1107/S1600536809050454/hy2256sup1.cif

Structure factors: contains datablocks I. DOI: 10.1107/S1600536809050454/hy2256Isup2.hkl

Additional supplementary materials:  crystallographic information; 3D view; checkCIF report

## Figures and Tables

**Table 1:** Selected bond lengths (Å)

Au—S1	2.3018 (12)
Au—P1	2.2473 (12)

**Table 2:** Hydrogen-bond geometry (Å, °)

*D*—H⋯*A*	*D*—H	H⋯*A*	*D*⋯*A*	*D*—H⋯*A*
C3—H3⋯O1^i^	0.94	2.52	3.365 (6)	150

Symmetry code: (i) 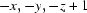.

**Fig. 1.**
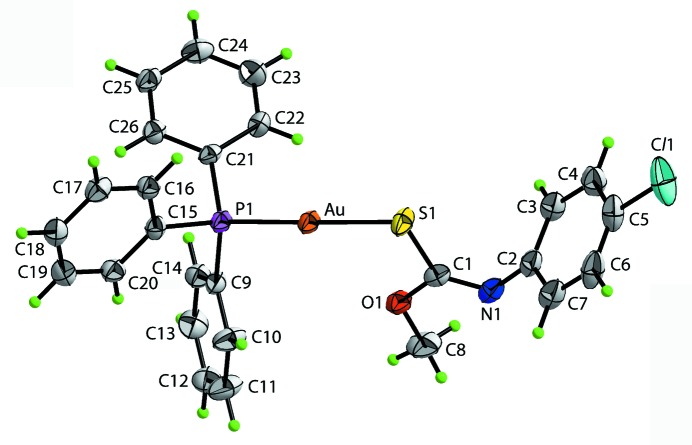
Molecular structure of the title compound. Displacement ellipsoids are drawn at the 50% probability level.

**Fig. 2.**
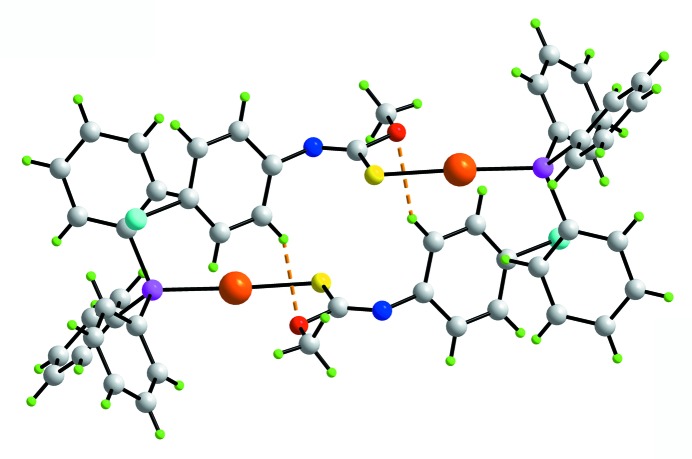
Supramolecular dimer in the title compound, mediated by C—H···O contacts (orange dashed lines). [Colour codes: Au orange; Cl cyan; S yellow; P pink; O red; N blue; C grey; and H green.]

## supplementary crystallographic information

### Comment

As a part of systematic studies of phosphinegold(I) thiocarbamides (Ho *et al.* 2006; Ho & Tiekink, 2007; Kuan *et al.*, 2008), the title compound, [(C_5_H_5_)_3_P]Au[SC(OMe)N(C_6_H_4_Cl-p)], was synthesized.

The thiocarbamide functions as a thiolate ligand as seen in the magnitudes of the C1—S1 and C1—N1 bond distances of 1.756 (5) and 1.271 (6) Å, respectively; the conformation about C1—N1 is *Z*. While the central SC(O)N chromophore is planar as seen in the S1—C1—N1—C2 and O1—C1—N1—C2 torsion angles of -0.8 (7) and -179.0 (4)°, respectively, the N-bound aryl ring is orthogonal to this plane as seen in the C1–N1–C2–C3 torsion angle of -76.6 (6)°. The thiocarbamide and phosphine ligands define an S, P donor set (Table 1 and Fig. 1). The deviation of the S1—Au—P1 angle [175.14 (5)°] from linearity is traced to the close approach of the O1 atom to the Au atom [2.882 (3) Å]. In the crystal structure, centrosymmetrically related molecules associate *via* C—H···O interactions (Table 2 and Fig. 2). The dimeric units formed are consolidated into the crystal structure by C—H···π interactions. The closest such contact occurs between C24—H24 and the ring centroid (Cg) of (C15—C20)^i^ [C24···*Cg*^i^ = 3.552 (6), H24···*Cg*^i^ = 2.73 Å, C24—H24···*Cg*^i^ = 146°, symmetry code: (i) -1+x, y, z].

### Experimental

The title compound was prepared following the standard literature procedure from the reaction of Ph_3_AuCl and MeOC(S)N(H)(C_6_H_4_Cl-*p*) in the presence of base (Hall *et al.*, 1993).

### Refinement

H atoms were geometrically placed (C—H = 0.94 and 0.97 Å) and refined as riding, with *U*_iso_(H) = 1.2(1.5 for methyl)*U*_eq_(C). The maximum and minimum residual electron density peaks of 1.17 and 0.62 e Å^-3^, respectively, were located 0.88 and 1.39 Å from the Au atom.

### Crystal data

**Table:**

[Au(C_8_H_7_ClNOS)(C_18_H_15_P)]	*F*(000) = 1280
*M**_r_* = 659.89	*D*_x_ = 1.804 Mg m^−^^3^
Monoclinic, *P*2_1_/*n*	Mo *K*α radiation, λ = 0.71069 Å
Hall symbol: -P 2yn	Cell parameters from 4456 reflections
*a* = 9.1781 (6) Å	θ = 2.3–25.0°
*b* = 17.5679 (12) Å	µ = 6.34 mm^−^^1^
*c* = 15.5384 (11) Å	*T* = 223 K
β = 104.156 (2)°	Block, colourless
*V* = 2429.3 (3) Å^3^	0.11 × 0.10 × 0.07 mm
*Z* = 4	

### Data collection

**Table:**

Bruker SMART APEX CCD diffractometer	5570 independent reflections
Radiation source: fine-focus sealed tube	4526 reflections with *I* > 2σ(*I*)
graphite	*R*_int_ = 0.042
φ and ω scans	θ_max_ = 27.5°, θ_min_ = 1.8°
Absorption correction: multi-scan (*SADABS*; Bruker, 2001)	*h* = −11→10
*T*_min_ = 0.363, *T*_max_ = 1.000	*k* = −22→22
17091 measured reflections	*l* = −20→14

### Refinement

**Table:**

Refinement on *F*^2^	Primary atom site location: structure-invariant direct methods
Least-squares matrix: full	Secondary atom site location: difference Fourier map
*R*[*F*^2^ > 2σ(*F*^2^)] = 0.029	Hydrogen site location: inferred from neighbouring sites
*wR*(*F*^2^) = 0.090	H-atom parameters constrained
*S* = 1.06	*w* = 1/[σ^2^(*F*_o_^2^) + (0.0462*P*)^2^*P*] where *P* = (*F*_o_^2^ + 2*F*_c_^2^)/3
5570 reflections	(Δ/σ)_max_ = 0.001
290 parameters	Δρ_max_ = 1.17 e Å^−^^3^
0 restraints	Δρ_min_ = −0.62 e Å^−^^3^

### Fractional atomic coordinates and isotropic or equivalent isotropic displacement parameters (Å^2^)

**Table:**

	*x*	*y*	*z*	*U*_iso_*/*U*_eq_	
Au	0.14582 (2)	0.203115 (10)	0.426452 (12)	0.02944 (8)	
Cl1	0.2539 (2)	−0.28046 (9)	0.29800 (15)	0.0761 (6)	
S1	0.19317 (16)	0.07649 (7)	0.40658 (8)	0.0372 (3)	
P1	0.08624 (13)	0.32352 (7)	0.45280 (8)	0.0265 (3)	
O1	0.2450 (4)	0.10253 (19)	0.5758 (2)	0.0342 (8)	
N1	0.2656 (4)	−0.0229 (2)	0.5444 (3)	0.0352 (9)	
C1	0.2379 (5)	0.0450 (3)	0.5172 (3)	0.0303 (10)	
C2	0.2605 (5)	−0.0827 (3)	0.4832 (3)	0.0350 (11)	
C3	0.1251 (5)	−0.1125 (3)	0.4349 (3)	0.0378 (12)	
H3	0.0343	−0.0911	0.4410	0.045*	
C4	0.1222 (6)	−0.1735 (3)	0.3779 (4)	0.0428 (13)	
H4	0.0304	−0.1931	0.3443	0.051*	
C5	0.2552 (7)	−0.2045 (3)	0.3715 (5)	0.0461 (14)	
C6	0.3906 (7)	−0.1768 (3)	0.4203 (5)	0.0507 (16)	
H6	0.4809	−0.1996	0.4153	0.061*	
C7	0.3939 (6)	−0.1165 (3)	0.4754 (4)	0.0434 (13)	
H7	0.4866	−0.0976	0.5084	0.052*	
C8	0.2730 (7)	0.0813 (4)	0.6684 (3)	0.0519 (15)	
H8A	0.1966	0.0456	0.6762	0.078*	
H8B	0.2699	0.1263	0.7040	0.078*	
H8C	0.3712	0.0576	0.6870	0.078*	
C9	0.1409 (5)	0.3443 (3)	0.5711 (3)	0.0295 (10)	
C10	0.2879 (6)	0.3272 (3)	0.6162 (4)	0.0466 (14)	
H10	0.3539	0.3047	0.5859	0.056*	
C11	0.3363 (7)	0.3434 (4)	0.7058 (4)	0.0535 (16)	
H11	0.4351	0.3316	0.7368	0.064*	
C12	0.2396 (7)	0.3768 (3)	0.7492 (4)	0.0471 (14)	
H12	0.2730	0.3885	0.8099	0.057*	
C13	0.0944 (6)	0.3936 (3)	0.7049 (4)	0.0436 (13)	
H13	0.0283	0.4159	0.7352	0.052*	
C14	0.0462 (6)	0.3773 (3)	0.6154 (3)	0.0326 (11)	
H14	−0.0528	0.3892	0.5848	0.039*	
C15	0.1717 (5)	0.3984 (2)	0.4012 (3)	0.0267 (9)	
C16	0.1530 (5)	0.3965 (3)	0.3101 (3)	0.0323 (10)	
H16	0.0989	0.3563	0.2770	0.039*	
C17	0.2129 (6)	0.4530 (3)	0.2673 (3)	0.0358 (11)	
H17	0.1990	0.4513	0.2053	0.043*	
C18	0.2926 (6)	0.5118 (3)	0.3152 (4)	0.0382 (12)	
H18	0.3328	0.5506	0.2862	0.046*	
C19	0.3131 (6)	0.5133 (3)	0.4061 (4)	0.0382 (12)	
H19	0.3684	0.5530	0.4393	0.046*	
C20	0.2532 (5)	0.4570 (3)	0.4485 (3)	0.0344 (11)	
H20	0.2681	0.4586	0.5105	0.041*	
C21	−0.1136 (5)	0.3406 (3)	0.4161 (3)	0.0259 (9)	
C22	−0.2118 (6)	0.2803 (3)	0.4140 (4)	0.0425 (13)	
H22	−0.1735	0.2315	0.4309	0.051*	
C23	−0.3653 (6)	0.2911 (3)	0.3874 (5)	0.0500 (15)	
H23	−0.4307	0.2499	0.3868	0.060*	
C24	−0.4224 (6)	0.3617 (3)	0.3618 (4)	0.0421 (13)	
H24	−0.5268	0.3689	0.3428	0.051*	
C25	−0.3268 (6)	0.4219 (3)	0.3641 (3)	0.0361 (11)	
H25	−0.3664	0.4705	0.3470	0.043*	
C26	−0.1724 (5)	0.4122 (3)	0.3913 (3)	0.0312 (10)	
H26	−0.1079	0.4540	0.3930	0.037*	

### Atomic displacement parameters (Å^2^)

**Table:**

	*U*^11^	*U*^22^	*U*^33^	*U*^12^	*U*^13^	*U*^23^
Au	0.03688 (12)	0.02458 (11)	0.02522 (11)	0.00819 (7)	0.00444 (8)	0.00001 (7)
Cl1	0.0969 (14)	0.0355 (8)	0.1131 (17)	−0.0060 (8)	0.0587 (13)	−0.0165 (9)
S1	0.0577 (8)	0.0266 (6)	0.0267 (6)	0.0125 (6)	0.0092 (6)	0.0013 (5)
P1	0.0296 (6)	0.0242 (6)	0.0250 (6)	0.0058 (5)	0.0050 (5)	−0.0002 (5)
O1	0.0384 (18)	0.0375 (19)	0.0250 (18)	0.0022 (15)	0.0045 (14)	0.0035 (15)
N1	0.032 (2)	0.037 (2)	0.037 (2)	0.0053 (17)	0.0106 (18)	0.0114 (19)
C1	0.028 (2)	0.036 (3)	0.027 (3)	0.0034 (19)	0.007 (2)	0.003 (2)
C2	0.035 (3)	0.028 (2)	0.043 (3)	0.003 (2)	0.013 (2)	0.012 (2)
C3	0.033 (3)	0.034 (3)	0.051 (3)	0.004 (2)	0.018 (2)	0.008 (2)
C4	0.038 (3)	0.035 (3)	0.058 (4)	−0.003 (2)	0.016 (3)	0.003 (3)
C5	0.056 (4)	0.026 (3)	0.065 (4)	0.001 (2)	0.030 (3)	0.006 (2)
C6	0.047 (3)	0.032 (3)	0.081 (5)	0.014 (2)	0.033 (3)	0.011 (3)
C7	0.032 (3)	0.040 (3)	0.061 (4)	0.006 (2)	0.019 (3)	0.015 (3)
C8	0.064 (4)	0.066 (4)	0.021 (3)	0.003 (3)	0.002 (3)	0.001 (3)
C9	0.038 (3)	0.024 (2)	0.025 (2)	0.0043 (19)	0.004 (2)	0.0020 (18)
C10	0.043 (3)	0.056 (4)	0.036 (3)	0.021 (3)	0.000 (2)	−0.006 (3)
C11	0.053 (4)	0.067 (4)	0.033 (3)	0.017 (3)	−0.005 (3)	−0.002 (3)
C12	0.067 (4)	0.043 (3)	0.025 (3)	0.001 (3)	−0.002 (3)	−0.003 (2)
C13	0.056 (3)	0.045 (3)	0.033 (3)	0.003 (3)	0.015 (3)	−0.005 (2)
C14	0.036 (3)	0.034 (3)	0.027 (3)	0.001 (2)	0.008 (2)	−0.001 (2)
C15	0.027 (2)	0.023 (2)	0.031 (2)	0.0050 (17)	0.0086 (19)	−0.0006 (19)
C16	0.036 (3)	0.029 (2)	0.030 (3)	0.003 (2)	0.004 (2)	−0.001 (2)
C17	0.040 (3)	0.039 (3)	0.029 (3)	0.006 (2)	0.011 (2)	0.003 (2)
C18	0.037 (3)	0.030 (3)	0.049 (3)	0.001 (2)	0.014 (2)	0.004 (2)
C19	0.038 (3)	0.033 (3)	0.043 (3)	−0.003 (2)	0.011 (2)	−0.003 (2)
C20	0.041 (3)	0.032 (3)	0.027 (3)	0.005 (2)	0.003 (2)	−0.003 (2)
C21	0.027 (2)	0.030 (2)	0.019 (2)	0.0047 (18)	0.0020 (18)	0.0013 (18)
C22	0.042 (3)	0.029 (3)	0.050 (3)	0.001 (2)	−0.003 (3)	0.011 (2)
C23	0.037 (3)	0.050 (4)	0.061 (4)	−0.008 (2)	0.008 (3)	0.011 (3)
C24	0.033 (3)	0.055 (4)	0.038 (3)	0.006 (2)	0.008 (2)	0.000 (3)
C25	0.041 (3)	0.032 (3)	0.033 (3)	0.012 (2)	0.004 (2)	−0.001 (2)
C26	0.033 (3)	0.030 (2)	0.029 (3)	0.0040 (19)	0.007 (2)	−0.002 (2)

### Geometric parameters (Å, °)

**Table:**

Au—S1	2.3018 (12)	C11—H11	0.9400
Au—P1	2.2473 (12)	C12—C13	1.374 (8)
Cl1—C5	1.755 (6)	C12—H12	0.9400
S1—C1	1.756 (5)	C13—C14	1.381 (7)
P1—C21	1.808 (4)	C13—H13	0.9400
P1—C15	1.815 (5)	C14—H14	0.9400
P1—C9	1.820 (5)	C15—C20	1.375 (6)
O1—C1	1.352 (6)	C15—C16	1.384 (6)
O1—C8	1.447 (6)	C16—C17	1.383 (7)
N1—C1	1.271 (6)	C16—H16	0.9400
N1—C2	1.409 (7)	C17—C18	1.375 (7)
C2—C3	1.387 (7)	C17—H17	0.9400
C2—C7	1.393 (7)	C18—C19	1.380 (7)
C3—C4	1.387 (8)	C18—H18	0.9400
C3—H3	0.9400	C19—C20	1.375 (7)
C4—C5	1.362 (8)	C19—H19	0.9400
C4—H4	0.9400	C20—H20	0.9400
C5—C6	1.377 (9)	C21—C22	1.385 (7)
C6—C7	1.357 (8)	C21—C26	1.386 (6)
C6—H6	0.9400	C22—C23	1.381 (8)
C7—H7	0.9400	C22—H22	0.9400
C8—H8A	0.9700	C23—C24	1.366 (7)
C8—H8B	0.9700	C23—H23	0.9400
C8—H8C	0.9700	C24—C25	1.369 (7)
C9—C14	1.363 (7)	C24—H24	0.9400
C9—C10	1.393 (7)	C25—C26	1.387 (7)
C10—C11	1.384 (8)	C25—H25	0.9400
C10—H10	0.9400	C26—H26	0.9400
C11—C12	1.370 (8)		
			
P1—Au—S1	175.14 (5)	C11—C12—C13	120.7 (5)
C1—S1—Au	100.35 (17)	C11—C12—H12	119.6
C21—P1—C15	104.8 (2)	C13—C12—H12	119.6
C21—P1—C9	107.1 (2)	C12—C13—C14	119.5 (5)
C15—P1—C9	105.0 (2)	C12—C13—H13	120.2
C21—P1—Au	111.82 (15)	C14—C13—H13	120.2
C15—P1—Au	116.79 (15)	C9—C14—C13	120.6 (5)
C9—P1—Au	110.73 (15)	C9—C14—H14	119.7
C1—O1—C8	116.4 (4)	C13—C14—H14	119.7
C1—N1—C2	120.3 (4)	C20—C15—C16	118.7 (4)
N1—C1—O1	120.3 (4)	C20—C15—P1	123.0 (4)
N1—C1—S1	126.9 (4)	C16—C15—P1	118.4 (3)
O1—C1—S1	112.7 (3)	C17—C16—C15	120.7 (5)
C3—C2—C7	118.8 (5)	C17—C16—H16	119.6
C3—C2—N1	121.6 (4)	C15—C16—H16	119.6
C7—C2—N1	119.4 (5)	C18—C17—C16	120.0 (5)
C4—C3—C2	120.8 (5)	C18—C17—H17	120.0
C4—C3—H3	119.6	C16—C17—H17	120.0
C2—C3—H3	119.6	C17—C18—C19	119.3 (5)
C5—C4—C3	118.5 (5)	C17—C18—H18	120.3
C5—C4—H4	120.7	C19—C18—H18	120.3
C3—C4—H4	120.7	C20—C19—C18	120.4 (5)
C4—C5—C6	121.6 (6)	C20—C19—H19	119.8
C4—C5—Cl1	119.2 (5)	C18—C19—H19	119.8
C6—C5—Cl1	119.2 (4)	C15—C20—C19	120.8 (5)
C7—C6—C5	120.1 (5)	C15—C20—H20	119.6
C7—C6—H6	120.0	C19—C20—H20	119.6
C5—C6—H6	120.0	C22—C21—C26	118.7 (4)
C6—C7—C2	120.2 (5)	C22—C21—P1	119.0 (4)
C6—C7—H7	119.9	C26—C21—P1	122.3 (4)
C2—C7—H7	119.9	C23—C22—C21	120.8 (5)
O1—C8—H8A	109.5	C23—C22—H22	119.6
O1—C8—H8B	109.5	C21—C22—H22	119.6
H8A—C8—H8B	109.5	C24—C23—C22	120.1 (5)
O1—C8—H8C	109.5	C24—C23—H23	119.9
H8A—C8—H8C	109.5	C22—C23—H23	119.9
H8B—C8—H8C	109.5	C23—C24—C25	119.7 (5)
C14—C9—C10	119.8 (5)	C23—C24—H24	120.1
C14—C9—P1	122.9 (4)	C25—C24—H24	120.1
C10—C9—P1	117.3 (4)	C24—C25—C26	120.8 (5)
C11—C10—C9	119.6 (5)	C24—C25—H25	119.6
C11—C10—H10	120.2	C26—C25—H25	119.6
C9—C10—H10	120.2	C21—C26—C25	119.7 (5)
C12—C11—C10	119.7 (5)	C21—C26—H26	120.1
C12—C11—H11	120.1	C25—C26—H26	120.1
C10—C11—H11	120.1		
			
C2—N1—C1—O1	−179.0 (4)	P1—C9—C14—C13	−178.5 (4)
C2—N1—C1—S1	−0.8 (7)	C12—C13—C14—C9	0.6 (8)
C8—O1—C1—N1	−4.9 (6)	C21—P1—C15—C20	110.4 (4)
C8—O1—C1—S1	176.7 (4)	C9—P1—C15—C20	−2.2 (4)
Au—S1—C1—N1	175.8 (4)	Au—P1—C15—C20	−125.2 (4)
Au—S1—C1—O1	−6.0 (3)	C21—P1—C15—C16	−69.1 (4)
C1—N1—C2—C3	−76.6 (6)	C9—P1—C15—C16	178.3 (4)
C1—N1—C2—C7	108.0 (5)	Au—P1—C15—C16	55.2 (4)
C7—C2—C3—C4	−1.8 (8)	C20—C15—C16—C17	−1.0 (7)
N1—C2—C3—C4	−177.2 (5)	P1—C15—C16—C17	178.5 (4)
C2—C3—C4—C5	1.2 (8)	C15—C16—C17—C18	0.3 (7)
C3—C4—C5—C6	0.1 (9)	C16—C17—C18—C19	0.5 (8)
C3—C4—C5—Cl1	−178.7 (4)	C17—C18—C19—C20	−0.6 (8)
C4—C5—C6—C7	−0.9 (9)	C16—C15—C20—C19	0.9 (7)
Cl1—C5—C6—C7	178.0 (4)	P1—C15—C20—C19	−178.6 (4)
C5—C6—C7—C2	0.3 (9)	C18—C19—C20—C15	−0.1 (8)
C3—C2—C7—C6	1.0 (8)	C15—P1—C21—C22	155.3 (4)
N1—C2—C7—C6	176.5 (5)	C9—P1—C21—C22	−93.6 (4)
C21—P1—C9—C14	−8.7 (5)	Au—P1—C21—C22	27.9 (4)
C15—P1—C9—C14	102.3 (4)	C15—P1—C21—C26	−25.9 (4)
Au—P1—C9—C14	−130.9 (4)	C9—P1—C21—C26	85.3 (4)
C21—P1—C9—C10	173.1 (4)	Au—P1—C21—C26	−153.3 (3)
C15—P1—C9—C10	−75.9 (5)	C26—C21—C22—C23	0.3 (8)
Au—P1—C9—C10	51.0 (5)	P1—C21—C22—C23	179.2 (5)
C14—C9—C10—C11	0.4 (9)	C21—C22—C23—C24	0.6 (10)
P1—C9—C10—C11	178.6 (5)	C22—C23—C24—C25	−1.1 (10)
C9—C10—C11—C12	−0.6 (10)	C23—C24—C25—C26	0.5 (8)
C10—C11—C12—C13	0.8 (10)	C22—C21—C26—C25	−0.8 (7)
C11—C12—C13—C14	−0.8 (9)	P1—C21—C26—C25	−179.7 (4)
C10—C9—C14—C13	−0.4 (8)	C24—C25—C26—C21	0.4 (7)

### Hydrogen-bond geometry (Å, °)

**Table:**

*D*—H···*A*	*D*—H	H···*A*	*D*···*A*	*D*—H···*A*
C3—H3···O1^i^	0.94	2.52	3.365 (6)	150

Symmetry codes: (i) −*x*, −*y*, −*z*+1.
